# Supplementary material for: The economics of accuracy for medical reasoning with large language models
Source: PLOS Digit Health. 2026 Sep 18;5(9):e0001182. doi: 10.1371/journal.pdig.0001182 (PMC13588378; doi:10.1371/journal.pdig.0001182)
Supplement: Supplementary file 1 [file pdig.0001182.s001.pdf]

S1 Appendix: Prompting strategies

S1.1 P0: Direct Answering

```
You are a medical expert. Answer the following multiple-choice question
    by selecting the correct option. Provide only the letter of your
    answer inside <answer></answer> tags.

Question: A 45-year-old woman presents with fatigue, weight gain, and
    cold intolerance. Laboratory studies show elevated TSH and low free
    T4. Which of the following is the most likely diagnosis?

A) Graves' disease
B) Hashimoto's thyroiditis
C) Subacute thyroiditis
D) Toxic multinodular goiter

<answer></answer>
```

S1.2 P1: Chain-of-Thought

```
You are a medical expert. Answer the following multiple-choice
    question. First, reason through the problem step-by-step inside
    <think></think> tags. Then, provide your final answer inside
    <answer></answer> tags.

Question: A 45-year-old woman presents with fatigue, weight gain, and
    cold intolerance. Laboratory studies show elevated TSH and low free
    T4. Which of the following is the most likely diagnosis?

A) Graves' disease
B) Hashimoto's thyroiditis
C) Subacute thyroiditis
D) Toxic multinodular goiter

<think>
[Reason through the clinical presentation, laboratory findings, and
    each answer choice step-by-step]
</think>

<answer></answer>
```

S1.3 P2: Self-Consistency with Majority Voting

The P2 strategy uses the same prompt as P1, executed five times independently at temperature 0.7.

S1.4 PCCR: Prompt-Chaining for Continuous Reflection

The initial prompt is the same prompt as P1. Below we provide the continuation prompt. The continuation prompt is repeated until the cumulative token count within

<think></think> tags meets or exceeds the specified budget (128, 256, 512, 768, or 1024 tokens). The final answer is extracted from the terminal response.

```
You are a medical expert continuing to reason through a multiple-choice
    question. Below is the original question and your reasoning so far.
    Continue reflecting on the problem: consider alternative
    interpretations, verify your logic, evaluate differential diagnoses
    you may have overlooked, and ensure your reasoning is complete. Add
    your continued reasoning inside <think></think> tags, then provide
    your final answer inside <answer></answer> tags.

Question: A 45-year-old woman presents with fatigue, weight gain, and
    cold intolerance. Laboratory studies show elevated TSH and low free
    T4. Which of the following is the most likely diagnosis?

A) Graves' disease
B) Hashimoto's thyroiditis
C) Subacute thyroiditis
D) Toxic multinodular goiter

Previous reasoning:
{PREVIOUS_REASONING}

Continue your analysis:
<think>
[Continue reasoning, verify conclusions, consider alternatives]
</think>

<answer></answer>
```
