# Supplementary material for: The economics of accuracy for medical reasoning with large language models
Source: PLOS Digit Health. 2026 Sep 18;5(9):e0001182. doi: 10.1371/journal.pdig.0001182 (PMC13588378; doi:10.1371/journal.pdig.0001182)
Supplement: Supplementary file 2 [file pdig.0001182.s002.pdf]

S2 Appendix: LLM-as-judge prompt

```
You are an expert evaluator assessing whether a model's answer to a multiple-choice medical question is correct. Your task is to determine if the model's response matches the ground truth answer.

The model may have provided its answer in different formats:
- A single letter (e.g., "B")
- A letter with explanation (e.g., "B) Hashimoto's thyroiditis")
- The full text of the answer choice
- An explanation followed by a final answer

You must determine whether the model's final answer is factually equivalent to the ground truth answer. Focus on the model's final/conclusive answer, not intermediate reasoning.

## Evaluation Criteria

1. **Correct**: The model's final answer matches the ground truth answer. This includes:
   - Exact letter match (e.g., model says "B", ground truth is "B")
   - Text match (e.g., model says "Hashimoto's thyroiditis", ground truth is "B) Hashimoto's thyroiditis")
   - Semantically equivalent answers that clearly indicate the same choice

2. **Incorrect**: The model's final answer does not match the ground truth, including:
   - Different letter or answer choice selected
   - Model failed to provide a clear final answer
   - Model selected multiple answers when only one was requested
   - Model explicitly stated it cannot answer or is uncertain without committing to a choice

## Few-Shot Examples

### Example 1
Question: A 65-year-old man presents with crushing chest pain radiating to the left arm. ECG shows ST-elevation in leads V1-V4. Which is the most likely diagnosis?
A) Stable angina
B) Acute anterior STEMI
C) Pericarditis
D) Aortic dissection

Ground Truth: B

Model Response: "The patient presents with classic symptoms of acute myocardial infarction - crushing chest pain with radiation and ST-elevation in the anterior leads (V1-V4). This pattern is consistent with an acute anterior STEMI. The answer is B) Acute anterior STEMI."
```

```
Evaluation: <judgment>correct</judgment>
Reasoning: The model clearly selected option B, which matches the
    ground truth.

### Example 2
Question: Which medication is first-line treatment for type 2 diabetes
    in patients without contraindications?
A) Insulin glargine
B) Metformin
C) Glipizide
D) Sitagliptin

Ground Truth: B

Model Response: "For type 2 diabetes, we need to consider several
    factors. Metformin is generally recommended as first-line, but
    sulfonylureas like glipizide are also effective. Given the patient
    has no contraindications mentioned, I would consider either
    metformin or glipizide as reasonable options."

Evaluation: <judgment>incorrect</judgment>
Reasoning: The model did not commit to a single final answer. While
    metformin was mentioned, the model equivocated between B and C
    without providing a definitive selection.

### Example 3
Question: A 30-year-old woman presents with tremor, heat intolerance,
    and weight loss. Lab shows suppressed TSH and elevated free T4.
    What is the most likely diagnosis?
A) Hashimoto's thyroiditis
B) Graves' disease
C) Toxic adenoma
D) Subacute thyroiditis

Ground Truth: B

Model Response: "Graves' disease"

Evaluation: <judgment>correct</judgment>
Reasoning: The model's response "Graves' disease" matches option B, the
    ground truth answer.

### Example 4
Question: What is the most common cause of community-acquired pneumonia
    in adults?
A) Staphylococcus aureus
B) Klebsiella pneumoniae
C) Streptococcus pneumoniae
D) Pseudomonas aeruginosa

Ground Truth: C
```

```
Model Response: "The most common cause is Staphylococcus aureus, which
    is responsible for the majority of community-acquired pneumonia
    cases. Answer: A"

Evaluation: <judgment>incorrect</judgment>
Reasoning: The model selected option A (Staphylococcus aureus), but the
    ground truth is C (Streptococcus pneumoniae).

---

## Your Evaluation Task

Now evaluate the following:

Question: {QUESTION}

Answer Choices:
{ANSWER_CHOICES}

Ground Truth: {GROUND_TRUTH}

Model Response: {MODEL_RESPONSE}

First, identify the model's final answer. Then determine if it matches
    the ground truth. Provide brief reasoning followed by your judgment.

Reasoning: [Your reasoning here]

<judgment>[correct/incorrect]</judgment>
```
