# Supplementary material for: The economics of accuracy for medical reasoning with large language models
Source: PLOS Digit Health. 2026 Sep 18;5(9):e0001182. doi: 10.1371/journal.pdig.0001182 (PMC13588378; doi:10.1371/journal.pdig.0001182)
Supplement: Supplementary file 3 [file pdig.0001182.s003.pdf]

S3 Appendix: Throughput Estimation Methodology

To estimate inference throughput for memory-bound decoding (batch size = 1), we utilized the Roofline model. For large language models, the generation phase is predominantly limited by memory bandwidth rather than compute capability. The theoretical maximum throughput R (tokens/second) is calculated as:

$$R = \frac{BW_{mem}}{S_{model}} = \frac{BW_{mem}}{N_{param} \cdot \beta_{eff}}$$

where:

- BW_{mem} is the effective memory bandwidth of the hardware (GB/s).
- N_{param} is the model parameter count (billions).
- β_{eff} is the effective size per parameter in bytes, accounting for precision and quantization overhead.

Precision Assumptions. We assumed the following effective memory footprints:

- **FP16 (Half Precision):** $\beta_{eff} = 2.0$ bytes/parameter.
- **INT4 (Quantized):** $\beta_{eff} \approx 0.65$ bytes/parameter (0.5 bytes for 4-bit weights + 0.15 bytes for group-wise quantization scales and zeros).

Hardware Specifications. Bandwidth values (BW_{mem}) were set as follows:

- **NVIDIA A100 (80GB SXM4):** 1,935 GB/s.
- **NVIDIA RTX 4090:** 1,008 GB/s.
- **Consumer CPU (DDR5 Dual-Channel):** $\sim$60 GB/s (estimated effective bandwidth for DDR5-6000).

Latency Bounds. For smaller models (e.g., 1B), the theoretical memory-bound throughput often exceeds the kernel launch latency limit. In these regimes, realized throughput is typically capped by compute overhead (kernel latency), often plateauing around 200–300 tokens/s on current GPU architectures regardless of available bandwidth.
