# Supplementary material for: The economics of accuracy for medical reasoning with large language models
Source: PLOS Digit Health. 2026 Sep 18;5(9):e0001182. doi: 10.1371/journal.pdig.0001182 (PMC13588378; doi:10.1371/journal.pdig.0001182)
Supplement: Supplementary file 4 [file pdig.0001182.s004.pdf]

S4 Appendix: Query Volume Estimation

To accurately amortize the fixed costs of fine-tuning, we estimate the annual query volume Q_{life} for a representative large academic health system. We assume the model is integrated into the electronic health record (EHR) workflow to assist with clinical documentation and decision support for every patient encounter.

Operational Baseline. We model a large integrated health network based on typical metrics for top-tier US hospital systems (e.g., systems with >2,000 beds):

- **Clinical Staff (N_{staff}):** 4,500 active providers (attending physicians, residents, and advanced practice providers).
- **Patient Encounters (R_{enc}):** Average of 18 encounters per provider per workday (outpatient visits, inpatient rounds, or procedure notes).
- **Operational Days (D_{ops}):** 230 clinical workdays per year per provider.

Query Generation Rate. We define the Query Rate (λ) as the number of model calls triggered per encounter. For a system providing automated note drafting or background clinical decision support (CDS), $\lambda \geq 1$.

$$Q_{annual} = N_{staff} \cdot R_{enc} \cdot D_{ops} \cdot \lambda$$

Table 23 calculates the projected volumes.

Table 23. Estimated annual query volume for a large hospital system.

**Parameter**	**Value**	**Rationale**
Clinical Providers (N_{staff})	4,500	Multi-hospital system (2,000+ beds)
Encounters/Day (R_{enc})	18	Avg. combined inpatient/outpatient load
Workdays/Year (D_{ops})	230	Accounting for vacation/admin time
**Total Encounters**	**18,630,000**	Baseline opportunity volume
Query Rate (λ)	1.0	1 query per encounter (e.g., Note Draft)
**Annual Query Volume**	**∼18.6 Million**	Q_{annual}

We approximate *20 million queries per year* as a reference query volume based on reasonable approximations. However, these estimates consider use at-scale with every encounter. Actual values during pilot phases or before broad adoption could be lower. Alternatively, multiple such systems serving different use-cases could result in multiple uses per encounter, leading to linear increases in query volume. Agentic orchestrations where services can query each other, could lead to far greater consumption.
