# Supplementary material for: The economics of accuracy for medical reasoning with large language models
Source: PLOS Digit Health. 2026 Sep 18;5(9):e0001182. doi: 10.1371/journal.pdig.0001182 (PMC13588378; doi:10.1371/journal.pdig.0001182)
Supplement: Supplementary file 5 [file pdig.0001182.s005.pdf]

S5 Appendix: RAG Cost Estimation Methodology

To determine a realistic retrieval cost per query (C_{RAG}), we utilize a Total Cost of Ownership (TCO) approach that distinguishes between marginal variable costs (OpEx) and amortized fixed infrastructure costs (CapEx + OpEx).

The total cost per query is modeled as:

$$C_{RAG} = C_{embed}(L_q) + \frac{C_{OpEx} + \frac{C_{CapEx}}{M}}{Q_{monthly}}$$

where:

- L_q is the average query length in tokens.
- $Q_{monthly}$ is the total number of queries processed per month.
- M is the amortization period in months (typically 36 for hardware).

1. Variable Embedding Cost (C_{embed}). This represents the API cost to embed the user's query vector.

$$C_{embed}(L_q) = \frac{L_q}{1,000,000} \cdot P_{emb}$$

where P_{emb} is the price per million tokens (e.g., $\sim$ \$0.02 for efficient embedding models). For a typical query ($L_q \approx 100$), this cost is negligible ($\approx$ \$0.000002).

2. Fixed Operational Expenses (C_{OpEx}). This includes monthly recurring costs for the vector database and retrieval infrastructure:

$$C_{OpEx} = P_{cloud\_db} + P_{hosting}$$

Example: A production-grade managed vector database (e.g., Pinecone, Weaviate) typically costs \$50–\$150/month for moderate distinct vector counts ($< 1M$).

3. Capital Expenses (C_{CapEx}). For on-premise or private cloud deployments utilizing dedicated hardware (e.g., GPU servers for local re-ranking or embedding):

$$C_{CapEx} = \text{Hardware Cost} + \text{Initial Setup Cost}$$

Sensitivity to Scale. RAG costs are dominated by vector database and GPU server cost for hosting embedding models. As shown below, low-volume applications face significantly higher unit costs due to unamortized fixed infrastructure:

$$C_{RAG} \approx \begin{cases} 600\mu\$ & \text{at } Q_{yearly} = 2,000,000 \text{ (Low Volume)} \\ 60\mu\$ & \text{at } Q_{yearly} = 20,000,000 \text{ (Medium Volume)} \\ 6\mu\$ & \text{at } Q_{yearly} = 200,000,000 \text{ (High Volume)} \end{cases}$$

Values shown in micro-dollars ($\mu\$ = 10^{-6}$ USD). At the expected query volume of 20 million queries per year, we should expect an amortized cost per query of $60\mu\$$ or \$0.00006. We round this up to \$0.0001 as the reference cost for our calculations.
