# Supplementary material for: The economics of accuracy for medical reasoning with large language models
Source: PLOS Digit Health. 2026 Sep 18;5(9):e0001182. doi: 10.1371/journal.pdig.0001182 (PMC13588378; doi:10.1371/journal.pdig.0001182)
Supplement: Supplementary file 6 [file pdig.0001182.s006.pdf]

S6 Appendix: Fine-tuning Cost Estimation

The total capital expenditure for fine-tuning (C_{FT}) is the sum of compute costs and data acquisition costs:

$$C_{FT}(S, \text{Quality}) = C_{compute}(S) + N_{examples} \cdot C_{data\_per\_example}$$

Compute Costs. Based on parameter-efficient fine-tuning (LoRA/QLoRA) benchmarks, compute costs are relatively minor compared to high-quality data costs. For a 27B model, fine-tuning requires approximately 4–12 hours on a single H100 or 8–24 hours on dual A100s, costing roughly \$40–\$192.

Data Costs. Data costs vary by orders of magnitude depending on the source:

- **Synthetic (LLM-generated):** \$0.01–0.05 per example.
- **Curated public/private:** \$0.10–0.50 per example (cleaning/formatting/annotation).
- **Expert-annotated (Clinical):** \$5–50 per example (physician or subject-matter-expert review).

Table 24 summarizes the total estimated fine-tuning costs.

Table 24. Estimated total fine-tuning costs (C_{FT}) by model size and data quality.

Model	Data Quality	Examples	Compute	Data Cost	Total C_{FT}
4B	Synthetic	10,000	\$25	\$250	\$275
4B	Curated public	10,000	\$25	\$2,500	\$2,525
4B	Expert-annotated	5,000	\$25	\$50,000	\$50,025
27B	Synthetic	10,000	\$100	\$250	\$350
27B	Curated public/private	10,000	\$100	\$2,500	\$2,600
27B	Expert-annotated	5,000	\$100	\$50,000	\$50,100

Amortization. The impact of C_{FT} on per-query cost depends heavily on query volume over the lifetime of the model (Q_{life}). Table 25 presents the amortized cost added to each query.

Table 25. Amortized fine-tuning cost per query by volume and data quality for 27B model. Values shown in micro-dollars ($\mu\$ = 10^{-6}$ USD). e.g., $35,000\mu\$ = \$0.035$.

Query Volume	Synthetic (\$350)	Curated (\$2,600)	Expert (\$50,100)
20,000	17,500μ\$	130,000μ\$	2,505,000μ\$ (\$5.01)
200,000	1,750μ\$	13,000μ\$	250,500μ\$ (\$0.50)
2,000,000	175μ\$	1,300μ\$	25,050μ\$ (\$0.05)
20,000,000	17.5μ\$	130μ\$	2,505μ\$ (\$0.005)
200,000,000	1.75μ\$	13μ\$	250.5μ\$ (\$0.0005)

At low volumes (<100k queries), expert-annotated fine-tuning adds significant cost (\$0.50–\$5.00 per query). At the reference query volume of 20 million queries per year, the amortized cost per query has a large range from $17.5\mu\$ - 2,505\mu\$$ based on data quality. Assuming that synthetic and curated dataset based fine-tuning can be performed by vendors, hospitals may only need to fine-tune on high-quality, expert-reviewed data. However, in this study, we use off-the-shelf fine-tuned models trained on curated data and, likely, synthetic data. Therefore, we choose the middle of the range as a reasonable per query amortization cost $\sim 1250\mu\$$ or \$0.00125. We round this to \$0.0013 for convenience.
