# Supplementary material for: The economics of accuracy for medical reasoning with large language models
Source: PLOS Digit Health. 2026 Sep 18;5(9):e0001182. doi: 10.1371/journal.pdig.0001182 (PMC13588378; doi:10.1371/journal.pdig.0001182)
Supplement: Supplementary file 7 [file pdig.0001182.s007.pdf]

# S7 Appendix: More example solutions to the optimization problem

### S7.0.1 Example 3: Latency-critical application

*Requirements:* $A_{min} = 70\%$, $L_{max} = 2s$, RAG available ($C = 1$)

We enumerate candidate configurations from Table 17 with $C = 1$ and achieving $\geq 70\%$ to populate Table 26. The optimal solution configuration is (4, 0, P2, 1) or Gemma-3 4B + P2 + RAG at 145$\mu$\$ per query and a latency of 1.8 seconds. Note that this latency can be reduced further and the accuracy improved by selecting the MedGemma 4B fine-tuned model, if the higher cost is still palatable.

**Table 26.** Candidate configurations for Example 3: Latency-critical application that meet the accuracy requirement. Values shown in micro-dollars ($\mu\$ = 10^{-6}$ USD).

| Config | Accuracy | Cost | Latency | Feasible? |
|---|---|---|---|---|
| (27, 1, P0, 1) | 74.7% | 1412$\mu$\$ | 3.7s | No (latency) |
| (27, 1, P1, 1) | 77.4% | 1430$\mu$\$ | 8.9s | No (latency) |
| (27, 0, P2, 1) | 70.1% | 250$\mu$\$ | 8.9s | No (latency) |
| (4, 1, P0, 1) | 73.1% | 1403$\mu$\$ | 0.9s | **Yes** |
| (4, 0, P2, 1) | 70.1% | 145$\mu$\$ | 1.8s | **Yes** |
| (4, 0, PCCR-128, 1) | 76.0% | 114.76$\mu$\$ | 2.76s | No (latency) |

### S7.0.2 Example 4: Budget-constrained application

*Requirements:* $A_{min} = 70\%$, $L_{max} = 10s$, RAG available, $C_{query} \leq 140\mu\$$

We enumerate candidate configurations from Table 17 with $C = 1$ and achieving $\geq 70\%$ to populate Table 27. The optimal solution configuration is (4, 0, PCCR-128, 1) or Gemma-3 4B + PCCR-128 + RAG at 114$\mu$\$ per query and a latency of 2.76 seconds. Low-cost requirements will favor generic models, as can be inferred from Fig 4

**Table 27.** Candidate configurations for Example 4: Latency-critical application that meet the accuracy requirement. Values shown in micro-dollars ($\mu\$ = 10^{-6}$ USD).

| Config | Accuracy | Cost | Latency | Feasible? |
|---|---|---|---|---|
| (27, 1, P0, 1) | 74.7% | 1412$\mu$\$ | 3.7s | No (cost) |
| (27, 1, P1, 1) | 77.4% | 1430$\mu$\$ | 8.9s | No (cost) |
| (27, 0, P2, 1) | 70.1% | 250$\mu$\$ | 8.9s | No (cost) |
| (4, 1, P0, 1) | 73.1% | 1403$\mu$\$ | 0.9s | No (cost) |
| (4, 0, P2, 1) | 70.1% | 145$\mu$\$ | 1.8s | No (cost) |
| (4, 0, PCCR-128, 1) | 76.0% | 114.76$\mu$\$ | 2.76s | **Yes** |
