# Supplementary material for: The economics of accuracy for medical reasoning with large language models
Source: PLOS Digit Health. 2026 Sep 18;5(9):e0001182. doi: 10.1371/journal.pdig.0001182 (PMC13588378; doi:10.1371/journal.pdig.0001182)
Supplement: Supplementary file 8 [file pdig.0001182.s008.pdf]

Response to Reviewers

The Economics of Accuracy for Medical Reasoning with Large Language Models

Manuscript revision — PLOS Digital Health

Kiran Bhattacharyya and Sreeram Kamabatulla

We thank the Editor and reviewers for their careful and constructive reading. The feedback has substantially improved the manuscript. Below we respond point-by-point. Each reviewer comment is reproduced in *brown italics*, followed by our response and a green **Changed** callout indicating exactly where in the revised manuscript the corresponding edit appears.

Tracking changes. We have prepared the revision so that a `latexdiff` against the original submission highlights all edits. In addition, the green callouts below name the portion where each change was made.

Response to the Editor

E1. Table-to-table inconsistencies

> *Resolve all table-to-table inconsistencies (Gemma-3 1B, P2 = 60.1% vs 63.1%).*

Response. We traced the discrepancy and found that the Gemma-3 1B P2 (with oracle context) value had drifted across tables. We reconciled it to **63.1%** which is the value reported in the self-consistency table, the optimization look-up table, and the Results text, and the value consistent with that model's own reported P2−P0 (+4.6) and P2−P1 (+4.5) deltas. We updated the PubMedQA accuracy table accordingly and corrected the "P2−P0 (with context)" column of the self-consistency-gain table, which had also drifted from its source table (the values now read +0.5, +10.1, +1.2, +9.8, +4.6, matching the accuracy table and the "+9.8 to +10.1" figures already stated in the text). We then performed a full consistency pass over every value appearing in more than one table.

> **Changed:** Table "Accuracy (%) on PubMedQA"; Table "Self-consistency (P2) accuracy gain"; Results § "Self-consistency (P2) provides robust gains with context." In-line code comments document each corrected cell.

E2. Statistical treatment / generalizability of the framework equations

> *Please expand the statistical treatment of the framework equations (fitting procedure, uncertainty on coefficients, goodness-of-fit), or reframe them as descriptive parameterizations rather than predictive models. Provide held-out validation, ideally cross-family.*

Response. We reframed the framework as an explicit *descriptive parameterization* rather than a predictive model. A new opening paragraph states that the equations summarize observed relationships, are not validated on held-out data, and carry coefficients that must be re-estimated for other models or settings; the transferable contribution is the structure and estimation method, not the specific numbers. For each fitted form we now state the fitting procedure (least-squares, or direct solution where parameters equal data points), the degrees of freedom, and goodness-of-fit: the baseline log fit reports mean absolute error ≈ 0.5 pp and pooled $R^2 = 0.95$, and the PCCR saturating-exponential reports MAE = $0.8\,\Delta\%$ and pooled $R^2 = 0.92$. Cross-family / held-out

validation would require new experiments; we name it as the single most important next step and note that qualitative patterns replicate across all three datasets as supportive but non-independent evidence.

> **Changed:** § "An Empirically Grounded Framework…": new "Interpretation and scope of the framework" paragraph; "Baseline accuracy" and PCCR-parameter paragraphs (now report MAE and R^2); Limitations and Future work (cross-family validation named as the priority next step).

E3. MedGemma-4B medical-exam inversion

Address the MedGemma-4B medical-exam inversion mechanistically.

Response. We added a mechanistic paragraph proposing that 4B-scale medical fine-tuning narrows the output distribution toward confident, template-like responses (evidenced by MedGemma's longer reasoning traces / fewer continuation rounds and MedGemma-4B's 13.2% answer-flip rate). On out-of-distribution Israeli board items this can lock the model onto confident-but-wrong templates, so added reasoning amplifies rather than corrects errors, while base Gemma-3 4B retains enough answer diversity to benefit. The clean P2 recovery indicates high-variance rather than systematically wrong reasoning. We present this as a hypothesis supported by, not proven by, the data.

> **Changed:** Results § "Performance on Medical Licensing Examination," new paragraph following the Gemma-3 4B vs MedGemma-4B reversal.

E4. Cost model vs. on-premise framing

Reconcile the cost model with the on-premise framing — consider either reworking it for hardware CapEx/OpEx or adjust the paper's framing.

Response. We restructured the cost narrative to lead with the total cost of ownership of *self-hosted* inference: memory footprint (e.g., a 27B model at ∼17 GB INT4 vs ∼54 GB FP16), throughput (tokens/second, memory-bandwidth-bound), and how context length scales KV-cache memory. Representative per-token prices are now explicitly framed as a convenience *proxy* for amortized self-hosted cost, not an endorsement of hosted APIs; the analysis depends only on the relative multipliers between strategies. The roofline throughput methodology and RAG / fine-tuning TCO (CapEx/OpEx, amortization) are cross-referenced from the appendices.

> **Changed:** § "The cost model": new on-premise TCO opening paragraph and proxy-pricing paragraph; caption of the per-token pricing table revised.

E5. Demand-side accuracy thresholds; MCQA transfer

Consider adding a demand-side discussion of accuracy thresholds for different clinical use cases, with explicit acknowledgment that MCQA may not transfer to open-ended clinical tasks.

Response. We added a Discussion subsection, "Matching accuracy thresholds to use cases," contrasting the supply-side framework with a demand-side view and providing an explicitly illustrative use-case to threshold table (drafting with review; retrieval-grounded decision support; autonomous

recommendation). We state that our equivalence points establish equal accuracy-per-dollar on a multiple-choice *proxy*, not clinical readiness, and that MCQA accuracy may not transfer to open-ended clinical tasks. The "practical significance for healthcare organizations" claim is now explicitly qualified where it appears.

> **Changed:** New § "Matching accuracy thresholds to use cases" with Table "Illustrative mapping…"; qualifying clause added in § "Revisiting the scaling paradigm."

E6. Clinical safety of answer flips and the overthinking penalty

Discuss clinical safety implications of answer flips and the overthinking penalty, including how a deployed system should mitigate them.

Response. We expanded Clinical implications with a "Safety of automated answer flips" paragraph naming the quantified hazard (up to 13.2% of correct answers flipping to incorrect under extended reasoning with context vs 1.8% without) and four data-grounded mitigations: cap the reasoning budget at $b^*(S)$ when context is reliable; prefer self-consistency over sequential reflection in RAG; use P2 answer-agreement as a calibration signal to route low-agreement cases to human review; and monitor flip rates as production safety telemetry.

> **Changed:** Results § "PCCR iteration characteristics" (flip-rate analysis); Discussion § "Clinical implications": new "Safety of automated answer flips" and "Other deployment factors" paragraphs.

E7. Multiple-comparisons correction and effect sizes

Apply multiple-comparisons correction and report effect sizes (Cohen's h or odds ratios) alongside p-values.

Response. Both added from existing data. We applied Benjamini–Hochberg FDR correction across the family of McNemar tests (25 tests; corrected threshold $p < 0.002$) and confirm that all comparisons reported as significant survive; we also note that our pre-existing rule of only comparing configurations separated by a full inflated 95% CI is intrinsically conservative. We added Cohen's h (with a definition and small/medium/large guidance) alongside percentage-point differences for the headline comparisons (e.g., CoT harm on Gemma-3 1B $h = 0.15$; PCCR MedGemma-27B gain $h = 0.48$; fine-tuning amplification at P2 $h = 0.35$; overthinking penalty $h = 0.16$).

> **Changed:** § Statistical analyses: new "Multiple comparisons" and "Effect sizes" paragraphs; Cohen's h inserted throughout Results and in the overthinking-penalty and optimal-reasoning table captions.

E8. PCCR continuation-prompt confound

Acknowledge and ideally ablate the PCCR continuation prompt as a possible confound (the instruction explicitly directs the model to second-guess, which may partly cause the "overthinking" effect).

Response. Acknowledged in Limitations. The continuation prompt does instruct the model to reconsider its reasoning, which could contribute to second-guessing. We argue this cannot be the sole driver, because the identical prompt improves accuracy without context and only harms it

with context. This implicates context availability, not wording, as the moderator. A controlled prompt-wording ablation is a new experiment; we name it as a prioritized next step.

> **Changed:** Discussion § Limitations: added a new paragraph beginning "Relatedly, the PCCR continuation prompt is itself a potential confound. . . "

E9. Tighten the Katz et al. dataset description

> *Tighten the Methods description of the Katz et al. medical exam dataset (country of origin, specialties, physician benchmark population, data-contamination considerations, etc.).*

Response. Rewritten with verified specifics: the 2022 Israeli board *residency* examinations administered by the Israeli Medical Association, five core specialties, benchmarked against the official scores of 849 practicing physicians with a 65% passing threshold and specialty-level median scores of 70–80%. We added an explicit data-contamination discussion: because these items are recent, non-US, and non-English in origin, they are less likely to appear in pretraining corpora, so we treat the dataset as a lower-contamination-risk generalization test while acknowledging we cannot fully exclude contamination.

> **Changed:** § Datasets: "Medical Licensing Examination Questions" bullet, substantially rewritten.

E10. Consolidated summary table; extraction-failure rates

> *Consider adding a consolidated summary results table before 2.6, and report the rate of empty-string regex extraction failures per model/prompt (Reviewer 1's point, also bears on small-model bias).*

Response. Both added. A consolidated summary table now appears immediately before the framework section, giving best configuration and P0 baseline range per dataset. We measured failed-extraction rates per model and prompting strategy (0.5–2% across models) and report them, with an upper bound showing that re-scoring every failed extraction as correct would raise the most-affected model's accuracy by at most 2 pp, leaving headline conclusions unchanged.

> **Changed:** New § "Consolidated summary of empirical results" within a new Table (placed before the framework section); § "Automated Accuracy Assessment" (failure-rate reporting and worst-case-bias bound).

E11. Ethics section

> *Please include an ethics section as per PLOS policy.*

Response. Added an Ethics statement covering: no human/animal subjects and use of only publicly available, de-identified benchmark data (no IRB or consent required); data and code availability; competing interests (disclosing the authors' Intuitive Surgical / SKA Labs affiliations); and a responsible-use statement reiterating that these systems are appropriate only for human-in-the-loop, clinician-supervised roles.

> **Changed:** New § "Ethics statement" before the References.

Response to Reviewer #1

We thank the reviewer for the generous framing ("a tour-de-force effort") and for three structural critiques that meaningfully sharpened the paper.

R1.1. Summary table before Section 2.6

> *Before Section 2.6, there should be a table summarizing the results.*

Response. Added. A consolidated summary table now precedes the framework section (see E10).

> **Changed:** New § "Consolidated summary of empirical results," Table `tab:results-summary`.

R1.2. Fitting approach, coefficient statistics, and generalizability of Section 2.6

> *Section 2.6 itself seems to posit a number of linear relationships fit to the data. While the general formulas seem well grounded, we are given no information on the regression-fitting approach, nor statistics around the coefficients found, nor goodness-of-fit...In particular, Section 2.7.5 seems to suggest that others should apply the author's approach, to other models and possibly other contexts. These constraints are not made explicit.*

Response. Addressed directly. Section 2.6 is now framed as a descriptive parameterization with the fitting procedure, degrees of freedom, and goodness-of-fit stated for each form (baseline: MAE $\approx$ 0.5 pp, $R^2 = 0.95$; PCCR: MAE $= 0.8\,\Delta\%$, $R^2 = 0.92$), and with an explicit statement that coefficients are dataset- and family-specific and must be re-estimated elsewhere. We revised the recommendations so the re-estimation requirement and limited-support caveat are stated up front (see E2).

> **Changed:** § "An Empirically Grounded Framework..." and "Interpretation and scope" paragraph; baseline and PCCR fit paragraphs.

R1.3. Limited generalizability; MCQA is low among healthcare AI needs

> *The Discussion does not clarify the limited generalizability of the results...the author states, "These equivalence points have practical significance for healthcare organizations". Yet multiple-choice question answering seems to be low on the list of needs or uses of (generative) AI for healthcare organizations, so the generalization needs to be supported.*

Response. We moved the generalizability caveats to the foreground and qualified the "practical significance" claims: the equivalence points establish equal accuracy-per-dollar on a multiple-choice proxy, not clinical readiness or transfer to open-ended tasks. The new demand-side subsection situates the results against actual clinical use cases.

> **Changed:** § "Revisiting the scaling paradigm" (qualifying clause); new § "Matching accuracy thresholds to use cases."

R1.4. The title promises "economics of accuracy" but never says what accuracy is worth

> *The article is titled, "The Economics of Accuracy," yet never addresses what level of accuracy is* worth *what level of investment. While reference is made to the 65% passing grade, I don't know if anyone would (or should) trust a mechanism that does not achieve higher than an 80% accuracy. . . none addresses what accuracy the environment needs.*

Response. This prompted the new subsection "Matching accuracy thresholds to use cases," which pairs the supply-side framework with a demand-side view: use cases carry different minimum-acceptable thresholds and error tolerances, mapped to the accuracy tiers our configurations reach. We engage the point directly that MCQA accuracy is necessary but not sufficient, the 65% board-pass mark is a floor not a target, and since our best configurations remain below the physician median, these systems belong in human-in-the-loop rather than autonomous roles, with the demand-side threshold (not maximum achievable accuracy) driving configuration choice.

Changed: New § "Matching accuracy thresholds to use cases", Table 22.

Response to Reviewer #2

We thank the reviewer for the detailed, section-by-section review and the recommendation of major revision. Every point is addressed below.

Abstract

> *1. The claim of "discovering equivalency points" is not formally defined (e.g., what margin of accuracy difference is considered equivalent).*

Response. The abstract now defines equivalence: two configurations are equivalent when their accuracies fall within one 95% confidence interval of each other (typically within 1–4 percentage points).

Changed: Abstract, "equivalency point" sentence.

> *2. The abstract does not report any measure of uncertainty or variability for the claimed accuracy improvements.*

Response. We added CI / significance information to the headline claims (e.g., the fine-tuning amplification "+4.6 to +15.7 pp, non-overlapping 95% CIs" and the PCCR gain "58.1% → 80.1%, $p < 10^{-5}$").

Changed: Abstract, sentences on fine-tuning amplification and PCCR.

> *3. The phrase "inflection points where context-grounding and test-time scaling used together lead to degrading performance" is vague; it does not specify the magnitude of degradation or under which conditions.*

Response. Replaced with magnitude and condition: with high-quality context, extended reasoning beyond roughly 128–256 tokens degrades accuracy by 7–13 percentage points (the "overthinking" penalty).

Changed: Abstract, "overthinking inflection" sentence.

Introduction

1. The introduction contains too much context which belongs to the method section.

Response. We have trimmed the Introduction and moved relevant sections to the Methods where they belong.

Changed: Introduction trimmed and content moved to Methods with further elaboration.

2. The introduction does not define what constitutes a "small" vs. "large" model in terms of parameter count for the purposes of their study.

Response. Added an explicit definition on first use: "small" $\leq$ 4B (1B, 4B) and "large" $\geq$ 27B, anchored to the $\sim$8B chain-of-thought threshold identified later.

Changed: Introduction, paragraph beginning "Despite the promise of these approaches. . . "

Methods — Models

1. No justification is given for why only the Gemma/MedGemma family is used, nor is there a discussion of potential architecture-specific biases.

Response. Added a paragraph giving the three reasons (scale span; shared base/fine-tuned architecture enabling confound-free measurement of fine-tuning; MedGemma as state-of-the-art open-weight medical model) and explicitly acknowledging that findings may be architecture-specific, cross-referencing the replication call in Limitations.

Changed: § Models, new paragraph beginning "We focused on the Gemma/MedGemma family for three reasons. . . "

2. The temperature settings (0.2 for deterministic conditions, 0.7 for self-consistency) are stated without any ablation or sensitivity analysis.

Response. We justify these as standard, literature-consistent choices (near-deterministic single-pass at 0.2; non-trivial sampling temperature required for self-consistency at 0.7) and acknowledge the absence of a temperature sweep as a limitation.

Changed: § Models, sentence following the temperature specification.

3. The medical licensing examination dataset is described as "publicly-available" but no details are given about potential data contamination.

Response. Addressed together with E9: the dataset description now specifies origin (2022 Israeli board residency exams) and includes an explicit contamination discussion, treating the dataset as a lower-contamination-risk generalization test.

Changed: § Datasets, "Medical Licensing Examination Questions" bullet.

4. No analysis of inter-annotator agreement or question difficulty is provided for any of the datasets.

Response. We note that difficulty and annotation quality are properties of these established third-party benchmarks (with the Katz exam providing an externally-validated difficulty anchor via physician performance) and acknowledge the absence of an independent inter-annotator / difficulty analysis as a limitation.

Changed: § Datasets, closing paragraph.

5. The token budget thresholds (128, 256, 512, 768, 1024) seem chosen arbitrarily; no rationale or pilot data is provided.

Response. We added a rationale: an approximately geometric-then-linear sweep spanning a single short reasoning pass (∼128 tokens) to ∼1,024 tokens, chosen to bracket the observed natural reasoning length (∼100–200 tokens) and to reveal both saturation and overthinking; a pragmatic rather than pilot-optimized choice.

Changed: § Prompting strategies, paragraph following the budget list.

6. "If extraction resulted in an empty string, the answer was scored as incorrect" — this may produce bias since small models are bad at strict structured output.

Response. We now report per-model / per-prompt failed-extraction rates (0.5–2%) and bound the worst-case bias (≤ 2 pp if every failed extraction were re-scored correct), and note that the LLM-as-judge adjudicates full-text answers rather than relying on strict label matching, so unstructured-but-correct outputs are not penalized.

Changed: § Automated Accuracy Assessment, opening paragraph.

7. McNemar's test is used for pairwise comparisons, but no correction for multiple comparisons (e.g., Bonferroni, FDR) is applied.

Response. Added Benjamini–Hochberg FDR correction (25 tests; corrected threshold $p < 0.002$); all but 2 reported significant results survive. We also make explicit that our comparison rule is already conservative (see E7).

Changed: § Statistical analyses, "Multiple comparisons" paragraph.

Results

1. The paper states that empty regex extraction was scored as incorrect, but never reports the rate of such failures per model or prompting strategy.

Response. Added; see Methods 6 above (0.5–2%, with worst-case-bias bound).

Changed: § Automated Accuracy Assessment.

2. For P2 (temperature 0.7, 5 samples), only the majority vote accuracy is reported. No standard deviation across independent runs of the 5-sample procedure is given.

Response. We now report the run-to-run standard deviation of P2 majority-vote accuracy ($\pm$0.5 pp), which is small relative to the reported confidence intervals, confirming that P2 accuracies are stable across repetitions.

Changed: § "Self-Consistency (P2) Agreement as a Calibration Signal," new opening paragraph.

3. The vertical axis of the line chart in Fig. 1 should be stretched, or the background dashed lines represented differently, because it is currently difficult to distinguish each line.

Response. The figure has been re-generated with y-axis bounds adjusted to the data bounds within each subpanel.

Changed: Figure 1

4. Only percentage point differences and p-values are given. Standardized effect sizes (e.g., Cohen's h, odds ratios) are absent.

Response. Added Cohen's h for the headline comparisons, computed from the reported proportions (see E7).

Changed: Results (throughout) and § Statistical analyses "Effect sizes."

5. Since the paper targets small models for local deployment, API pricing is less relevant. Instead, discuss deployment memory requirements, tokens per second, and the relationship between context length and memory usage.

Response. We restructured the cost narrative to foreground exactly these quantities: memory footprint at INT4/FP16, throughput in tokens/second, and KV-cache growth with context length. This surfaces the material into the main text and demotes per-token pricing to a proxy (see E4).

Changed: § "The cost model," new on-premise TCO paragraphs; Table 16 referenced in main text.

Discussion

1. The Discussion focuses entirely on accuracy, cost, and latency. It does not consider explainability, calibration, hallucination rates, or the risk of automated answer flips (13.2% flip rate in some conditions).

Response. Addressed with E6. Clinical implications now foregrounds the answer-flip hazard and mitigations, and adds a paragraph acknowledging explainability, calibration, and hallucination as essential deployment factors, noting that the P2 answer-agreement signal provides a partial, training-free calibration estimate.

Changed: § Clinical implications: "Safety of automated answer flips" and "Other deployment factors" paragraphs.

2. The paragraph beginning "We evaluated four prompting strategies" needs more citations because some of the inferences seem too arbitrary and lack support.

Response. We added inline citations at each analogy (tree-of-thought, self-reflection, multi-agent debate) and softened the claims to conceptual parallels rather than equivalences, noting that PCCR does not implement the explicit search / critic / multi-agent machinery of those methods.

> **Changed:** § Limitations, paragraph beginning "We evaluated four prompting strategies..."

> *3. The validation of the LLM-as-judge shows near-perfect agreement, with only two discrepancies. Was it a random error, or does it reveal a systematic bias in the LLM judge?*

Response. We now report that the two disagreements fell in opposite directions (one false-positive, one false-negative), consistent with random, borderline free-text adjudication rather than a systematic directional bias, and note that our CI-inflation procedure already propagates the judge's 1% error rate into every reported interval.

> **Changed:** § "Human Validation of the LLM-as-judge."

We are grateful for the reviewers' time and believe the manuscript is substantially stronger for their input. We would be happy to make any further changes the Editor considers necessary.
