# Supplementary material for: The economics of accuracy for medical reasoning with large language models
Source: PLOS Digit Health. 2026 Sep 18;5(9):e0001182. doi: 10.1371/journal.pdig.0001182 (PMC13588378; doi:10.1371/journal.pdig.0001182)
Supplement: Peer review history

# Peer review: Decision Letter 0

**DOI:** 10.1371/journal.pdig.0001182.r001

**Authors:** Dominic Marshall, Amara Tariq

22 May 2026

Response to Reviewers Revised Manuscript with Track Changes Manuscript **Journal Requirements:**

1. Please ensure that the CRediT author contributions listed for every co-author are completed accurately and in full.

At this stage, the following Authors require contributions: Kiran Bhattacharyya. Please ensure that the full contributions of each author are acknowledged in the "Add/Edit/Remove Authors" section of our submission form.

The list of CRediT author contributions may be found here: INSERT LINK

https://journals.plos.org/digitalhealth/s/authorship#loc-author-contributions

2. Please send a completed 'Competing Interests' statement, including any COIs declared by your co-authors. If you have no competing interests to declare, please state "The authors have declared that no competing interests exist". Otherwise please declare all competing interests beginning with the statement "I have read the journal's policy and the authors of this manuscript have the following competing interests:"

3. We ask that a manuscript source file is provided at Revision. Please upload your manuscript file as a .doc, .docx, .rtf or .tex.

4. Please provide separate figure files in .tif or .eps format.

For more information about figure files please see our guidelines:  LINK

https://journals.plos.org/digitalhealth/s/figures

https://journals.plos.org/digitalhealth/s/figures#loc-file-requirements

**Additional Editor Comments (if provided):**

PLease expand the statistical treatment of the framework equations (fitting procedure, uncertainty on coefficients, goodness-of-fit), or reframe them as descriptive parameterizations rather than predictive models. Provide held-out validation, ideally cross-family.

Address the MedGemma-4B medical-exam inversion mechanistically.

Reconcile the cost model with the on-premise framing conisder either reworking it for hardware CapEx/OpEx or adjust the paper's framing.

Consider adding a demand-side discussion of accuracy thresholds for different clinical use cases, with explicit acknowledgment that MCQA may not transfer to open-ended clinical tasks.

Discuss clinical safety implications of answer flips and the overthinking penalty, including how a deployed system should mitigate them.

Apply multiple-comparisons correction and report effect sizes (Cohen's h or odds ratios) alongside p-values.

Acknowledge and ideally ablate the PCCR continuation prompt as a possible confound (the instruction explicitly directs the model to second-guess, which may partly cause the "overthinking" effect).

Tighten the Methods description of the Katz et al. medical exam dataset (country of origin, specialties, physician benchmark population, data-contamination considerations etc).

Consider adding a consolidated summary results table before 2.6, and report the rate of empty-string regex extraction failures per model/prompt (Reviewer 1's point, also bears on small-model bias).

**Reviewers' Comments:**

**Comments to the Author**

1. Does this manuscript meet PLOS Digital Health’s publication criteria?

Reviewer #1: Yes

Reviewer #2: Yes

**********

2. Has the statistical analysis been performed appropriately and rigorously?

Reviewer #1: No

Reviewer #2: Yes

**********

3. Have the authors made all data underlying the findings in their manuscript fully available (please refer to the Data Availability Statement at the start of the manuscript PDF file)?

The PLOS Data policy

Reviewer #1: Yes

Reviewer #2: Yes

**********

4. Is the manuscript presented in an intelligible fashion and written in standard English?

Reviewer #1: Yes

Reviewer #2: Yes

**********

Reviewer #1: This is a tour-de-force effort to work out a complex ecology around LLMs and medical question answering.

Before Section 2.6, there should be a table summarizing the results.

Section 2.6 itself seems to posit a number of linear relationships fit to the data. While the general formulas seem well grounded, we are given no information on the regression-fitting approach, nor statistics around the coefficients found, nor goodness-of-fit. The main concern is that the formulas are fit to these data and so have unclear generalizability. In particular, Section 2.7.5 seems to suggest that others should apply the author’s approach, to other models and possibly other contexts. These constraints are not made explicit in that section.

The Discussion does not clarify the limited generalizability of the results, despite the Limitations section. For instance, the author states, “These equivalence points have practical significance for healthcare organizations”. Yet multiple-choice question answering seems to be low on the list of needs or uses of (generative) AI for healthcare organizations, so the generalization needs to be supported. The authors continue to make statements about the (general) use of their results, without making this question of generalizability front and center.

Finally, the article is titled, “The Economics of Accuracy,” yet never addresses what level of accuracy is *worth* what level of investment. While reference is made to the 65% passing grade, I don’t know if anyone would (or should) trust a mechanism that does not achieve higher than an 80% accuracy. While the formulas are great (although possibly not generalizable) in the several tradeoffs discussed, none addresses what accuracy the environment needs.

Reviewer #2: This is a methodologically rigorous and highly valuable study. The authors systematically evaluate trade-offs between model scale, fine-tuning, test-time scaling, and retrieval augmentation across multiple medical QA datasets. The introduction of PCCR is clever, the empirical coverage is extensive, and the derived framework for cost–accuracy–latency optimization is both novel and practically useful.

However, a few minor issues remain that should be addressed before acceptance:

Abstract

1. The claim of “discovering equivalency points” is not formally defined in the abstract (e.g., what margin of accuracy difference is considered equivalent).

2. The abstract does not report any measure of uncertainty or variability for the claimed accuracy improvements (e.g., confidence intervals or statistical significance).

3. The phrase “inflection points where context-grounding and test-time scaling used together leadto degrading performance” is vague; it does not specify the magnitude of degradation or under which conditions.

Introduction

1. The introduction contain too much context which belongs to method section

2. The introduction does not define what constitutes a “small” vs. “large” model in terms of parameter count for the purposes of their study.

Methods

Section 1.1 (Models)

1. No justification is given for why only the Gemma/MedGemma family is used, nor is there a discussion of potential architecture-specific biases.

2. The temperature settings (0.2 for deterministic conditions, 0.7 for self-consistency) are stated without any ablation or sensitivity analysis.

3.The medical licensing examination dataset is described as “publicly-available” but no details are given about potential data contamination (i.e., whether models were pre-trained on these exact questions).

4. No analysis of inter-annotator agreement or question difficulty is provided for any of the datasets.

5. The token budget thresholds (128, 256, 512, 768, 1024) are seems chosen arbitrarily; no rationale or pilot data is provided to justify these specific values.

6. The paper states that “if extraction resulted in an empty string, the answer was scored as incorrect” – this may produce bias since small model are bad at strict structured output

7. McNemar’s test is used for pairwise comparisons, but no correction for multiple comparisons (e.g., Bonferroni, FDR) is applied across the large number of reported comparisons.

Results

1.The paper states that empty regex extraction was scored as incorrect, but never reports the rate of such failures per model or prompting strategy. This could bias results against models that produce less structured outputs.

2.For P2 (temperature 0.7, 5 samples), only the majority vote accuracy is reported. No standard deviation across independent runs of the 5-sample procedure is given, so sampling variability is unknown.

3. The vertical axis of the line chart in Fig. 1 should be stretched or the background dashed lines should be represented in another form, because it is currently somewhat difficult to distinguish each line.

4. Only percentage point differences and p-values are given. Standardized effect sizes (e.g., Cohen’s h, odds ratios) are absent, making it difficult to assess practical significance across different baseline accuracies.

5.Since the paper targets small models for local deployment, API pricing is less relevant. Instead, discuss deployment memory requirements, tokens per second, and the relationship between context length and memory usage.

Discussion

1. The Discussion focuses entirely on accuracy, cost, and latency. It does not consider other critical deployment factors such as explainability, calibration, hallucination rates, or the risk of automated answer flips (as seen in PCCR). A clinical system that flips from correct to incorrect after more “thinking” (13.2% flip rate in some conditions) could be dangerous. This risk is not discussed in the clinical implications.

2. This paragraph, which begins with "We evaluated four prompting strategies," needs more citations because some of the inferences seem too arbitrary and lack support.

3. The validation of the LLM-as-judge shows near-perfect agreement), with only two discrepancy between the human evaluator and the LLM.Was it a random error, or does it reveal a systematic bias in the LLM judge.

Overall, this paper has strong potential, but the authors need to address the methodological gaps, improve transparency in reporting, and expand the discussion to cover clinically relevant risks. So i recommend a major revision to this paper.

**********

what does this mean?). If published, this will include your full peer review and any attached files.

**Do you want your identity to be public for this peer review?**  If you choose “no”, your identity will remain anonymous but your review may still be made public.

For information about this choice, including consent withdrawal, please see our Privacy Policy

Reviewer #1: No

Reviewer #2: **Yes**:  Yuexiong Yi

**********

**Figure resubmission:**

**Reproducibility:**

To enhance the reproducibility of your results, we recommend that authors of applicable studies deposit laboratory protocols in protocols.io, where a protocol can be assigned its own identifier (DOI) such that it can be cited independently in the future. Additionally, PLOS ONE offers an option to publish peer-reviewed clinical study protocols. Read more information on sharing protocols at https://plos.org/protocols?utm_medium=editorial-email&utm_source=authorletters&utm_campaign=protocols

# Author comment: Author response to Decision Letter 1

**DOI:** 10.1371/journal.pdig.0001182.r002

28 Jul 2026

Attachment Submitted filename: Econ-accuracy-medical-llms-response-to-reviewers.pdf

# Editor report: Decision Letter 1

**DOI:** 10.1371/journal.pdig.0001182.r003

**Authors:** Dominic Marshall, Amara Tariq

26 Aug 2026

The Economics of Accuracy for Medical Reasoning with Large Language Models

PDIG-D-25-01219R1

Dear Mr. Bhattacharyya,

We are pleased to inform you that your manuscript 'The Economics of Accuracy for Medical Reasoning with Large Language Models' has been provisionally accepted for publication in PLOS Digital Health.

Before your manuscript can be formally accepted you will need to complete some formatting changes, which you will receive in a follow-up email from a member of our team.

Please note that your manuscript will not be scheduled for publication until you have made the required changes, so a swift response is appreciated.

IMPORTANT: The editorial review process is now complete. PLOS will only permit corrections to spelling, formatting or significant scientific errors from this point onwards. Requests for major changes, or any which affect the scientific understanding of your work, will cause delays to the publication date of your manuscript.

If your institution or institutions have a press office, please notify them about your upcoming paper to help maximize its impact. If they'll be preparing press materials, please inform our press team as soon as possible -- no later than 48 hours after receiving the formal acceptance. Your manuscript will remain under strict press embargo until 2 pm Eastern Time on the date of publication. For more information, please contact digitalhealth@plos.org.

Thank you again for supporting Open Access publishing; we are looking forward to publishing your work in PLOS Digital Health.

Best regards,

Dominic Marshall

Guest Editor

PLOS Digital Health

***********************************************************

**Additional Editor Comments (if provided):**

Thank you for a thorough and responsive revision which I am pleased to accept. Two small points, neither of which requires any change to the manuscript.

First, the response to Reviewer 2 (Methods, point 7) states that following Benjamini–Hochberg FDR correction "all but 2 reported significant results survive". This appears to be the Bonferroni result (23 of 25 at the 0.002 threshold) rather than the FDR result. The manuscript itself is correct and distinguishes the two clearly, reporting that all comparisons survive Benjamini–Hochberg and that 23 of 25 additionally survive the stricter Bonferroni threshold. Relatedly, both this response and the response to Editor point 7 describe 0.002 as the "corrected threshold" for Benjamini–Hochberg, whereas 0.002 is 0.05/25, the Bonferroni threshold; Benjamini–Hochberg uses rank-dependent thresholds. No manuscript change is needed, but should you opt in to publish the peer review history, you may wish to correct the response document so the published record is internally consistent.

Second, a minor typographical point to update when you get the proofs. In the results section on majority voting, there is a doubled comma in "(+2.6 ∆%, , p < 10⁻²)".
